# Prognostic factors in patients undergoing early-start peritoneal dialysis within 24 h after catheter insertion

**DOI:** 10.1590/1414-431X20188055

**Published:** 2019-03-25

**Authors:** Hong Ying Jiang, Dan Ju Huang, Yi Hua Bai, Ji Sai Li, Hong Yan Pi, Jing Chen, Luo Hua Li, Jing Li

**Affiliations:** Department of Nephrology, The Second Affiliated Hospital, Kunming Medical University, Kunming, Yunnan Province, China

**Keywords:** Peritoneal dialysis, Early-start peritoneal dialysis, Survival rates, End stage renal disease, Dialysis

## Abstract

This study aimed to investigate the clinical characteristics, prognosis, and factors for survival of patients who underwent early-start peritoneal dialysis (PD) within 24 h after catheter insertion three years after PD. This study was conducted from January 1, 2013 to December 31, 2017. All adult patients who were diagnosed with end-stage renal disease (ESRD) and underwent PD for the first time within 24 h after catheter insertion in our hospital were included. All patients with PD were followed-up until they withdrew from PD, switching to hemodialysis, were transferred to other medical centers, underwent renal transplantation, died or were lost to follow-up, or continued to undergo dialysis until the end of the study period. The follow-up observation lasted three years. The number of eligible patients was 110, and switching to hemodialysis and death were the main reasons for patients to withdraw from PD. The 1-, 2-, and 3-year technical survival rates of patients were 89.1, 79.1, and 79.1% respectively, while the 1-, 2- and 3-year survival rates were 90, 81.8, and 81.8%, respectively. The Charlson comorbidity index, age, hemoglobin, serum albumin, diabetic nephropathy, chronic glomerulonephritis, and hypertensive renal damage were independent risk factors that affected the prognosis of PD patients. Under the condition of ensuring the quality of the PD catheter insertion, early-start PD within 24 h after catheter insertion is a safe treatment approach for ESRD patients.

## Introduction

With the increase in population and population aging, the incidences of chronic kidney disease (CKD) and end-stage renal disease (ESRD) have also increased. A multicenter study of CKD epidemiology in China in 2012 revealed that the prevalence of CKD among Chinese adults has reached 10.8%, and its incidence in the southwestern region where Kunming is located has reached 18.3% (1). In 2017, the prevalence of CKD was 39.1% (2) in patients who were engaged in the management program of chronic diseases in Kunming.

CKD slowly progresses and deteriorates into ESRD, and requires dialysis therapy. Compared with hemodialysis (HD), peritoneal dialysis (PD) is safe, reliable, and inexpensive, and can effectively protect residual renal function and stabilize hemodynamics. Therefore, PD has been widely used worldwide. It has been considered that the incidence of catheter-related complications is higher at earlier PD (3,4), which may cause technical failure of PD. Furthermore, a number of related reports have suggested that PD should begin 14 days after insertion of the peritoneal catheter. However, this restricts the application of PD in emergency dialysis.

Kunming is located in the southwest plateau region, and a variety of ethnic minorities gather in this location. However, economic development is poor and the incidence of CKD is high. Furthermore, in this region, most ESRD patients do not know about the disease, and the majority of ESRD patients are critically ill at first visit and require immediate dialysis. In addition, due to economic constraints, temporary HD cannot be used for transitional treatment after PD catheter insertion, and emergency PD treatment must be started as soon as possible after catheter insertion. In this study, although all patients accepted PD treatment within 72 h after catheter insertion, we also proposed early-start PD because of the term of urgent-start PD reported by Blake et al. (5).

However, the shortest time after catheter insertion for safe PD remains to be determined. In the present study, the clinical features and factors affecting the survival and prognosis of patients with early-start PD who received three years of dialysis in our hospital were analyzed and the feasibility of PD within 24 h after catheter insertion is discussed in order to provide a basis for the timing of clinical PD.

## Material and Methods

### Study subjects

Subjects were enrolled in the present study from January 1, 2013 to December 31, 2017. A total of 110 adult ESRD patients were admitted in the Department of Nephrology of the Second Affiliated Hospital of Kunming Medical University and diagnosed with ESRD. These patients received early-start PD within 24 h after first catheter insertion. All catheters used for PD were surgically implanted by kidney specialists at our center. All patients were followed-up for at least three years after catheter insertion. Follow-up termination events included withdrawal from PD, switching to hemodialysis, renal transplantation, being transferred to other medical centers, or lost to follow-up.

The endpoint event of the survival rate calculation was death; the survival rate analysis did not include patients who had failure of renal transplantation and PD, and were transferred to other medical centers. The endpoint events of the technical survival rate calculation included the continuation or termination of PD. However, the survival or death of patients at that time-point was not taken into account. Failure of PD included failure of ultrafiltration leading to HD or other mechanical problems.

Inclusion criteria were: 1) patients diagnosed with ESRD (GFR <15 mL·min^−1^·(1.73 m^2^)^−1^); 2) patients who had PD for the first time within 24 h after catheter insertion; 3) patients who had a follow-up duration of ≥3 months; 4) patients who were ≥18 years old. Exclusion criteria were: 1) patients without complete medical records or follow-up data; 2) patients with malignant tumors; 3) patients who recently underwent an abdominal operation (<30 days), which is the absolute contraindication of PD; 4) patients who underwent multiple abdominal operations (>2 times).

### Dialysis method

In the present study, all selected patients were implanted with a PD catheter through surgical incision by surgeons in the Department of Nephrology in our center under local anesthesia. The catheter was a double polyester sleeve straight Tenckhoff dialysis catheter with a double dialysis connection system produced by Baxter (USA). Patients were treated with early-start PD within 24 h after catheter insertion, and the specific dialysis plan was adjusted according to the clinical situation of the PD patient. After the insertion of the catheter, PD began at a rate of 1 L/time. Then, the liquids were exchanged 4–6 times a day, and the rate was gradually increased to 2 L/time. After two weeks, continuous ambulatory peritoneal dialysis was carried out. Standard lactate dialysate (Baxter) was chosen, and the dialysate glucose concentration was 1.5 and 2.5%. Ion composition was: sodium, 132 mmol/L; calcium, 1.77 mmol/L; magnesium, 0.25 mmol/L; chlorine, 96 mmol/L; lactate, 40 mmol/L. In the present study, all subjects provided written informed consent prior to enrollment into the present study and the study was approved by the Ethics Committee of our hospital.

### Data collection

The data was obtained through medical information registration and telephone follow-ups. The age, gender, main causes of ESRD, blood pressure, height, weight, body mass index (BMI), biochemical examination, and the Charlson comorbidity index (CCI) scores of urgent-start PD patients were recorded. CCI is a reliable stratification method proposed by Charlson et al. (6), which can be used to predict the risk of death of a disease. On the basis of 19 related diseases (hypertension, diabetes, cardiovascular and cerebrovascular diseases, pulmonary diseases, liver diseases, tumors, leukemia, lymphoma, acquired immunodeficiency syndrome, etc.), the score was calculated according to the weight of the corresponding disease, and the sum is the final score of the comorbidities of patients. BMI (kg/m^2^) was divided into 4 levels, according to World Health Organization BMI classification standards (7): underweight: BMI <18.5; normal weight: BMI within 18.5–25.0; overweight: BMI within 25–30; obesity: BMI >30.

### Statistical analysis

Data were statistically analyzed using SPSS 21.0 statistical software. Normally distributed measurement data are reported as means±SD; non-normally distributed measurement data are reported as median (and interquartile range); count data are reported as frequency (rate/proportion). Measurement data in normal distribution were compared using *t*-test, while measurement data in non-normal distribution were compared using rank-sum test. Count data were compared using chi-squared test. The Kaplan-Meier method was used for the survival analysis and statistics, in which the survival rate and technical survival rate were calculated, and survival curves were drawn. The survival curves were compared using the log-rank method. Factors that were statistically significant in the univariate COX proportional hazards regression models (P<0.05) were included in the multivariate COX regression model in order to analyze the independent risk factors that affected the prognosis. P<0.05 was considered statistically significant.

## Results

### Clinical characteristics and outcomes

A total of 110 patients were included in the present study. Among these patients, 95 patients (86.4%) were <65 years old and 15 patients (13.6%) were ≥65 years old. The follow-up duration for PD was 36 months, the mean survival time was 32.94±7.65 months, and the BMI ranged within 14.67-38.70. The biochemical indexes of PD patients before dialysis are presented in Table 1.

**Table 1:** Demographic characteristics of patients treated with urgent-start peritoneal dialysis.

Items	Results
Male (%)	63 (57.3)
Age (years)	50.57±12.38
Primary Diseases (n, %)	
Chronic glomerulonephritis	48 (43.6)
Hypertensive renal damage	35 (31.8)
Diabetic nephropathy	20 (18.2)
Other	7 (6.4)
BMI (kg/m^2^) (n, %)	
Normal weight	75 (68.2)
Underweight	16 (14.5)
Overweight	15 (13.6)
Obesity	4 (3.6)
CCI (n, %)	
1	1 (0.9)
2	60 (54.5)
3	32 (29.1)
4	13 (11.8)
5	3 (2.7)
6	1 (0.9)
Systolic blood pressure (mmHg) (median, IQR)	140 (123,155)
Diastolic blood pressure (mmHg) (median, IQR)	80 (70,96.5)
NEUT (×10^9^/L)	4.54±2.27
LYMPH (×10^9^/L)	1.31±0.45
HGB (g/L) (median, IQR)	85 (73,97)
ALB (g/L) (median, IQR)	29.3 (25.5,33.6)
CK-MB (ng/mL)	13.77±8.37
TC (mmol/L)	4.40±1.19
GLU (mmol/L)	5.16±1.94
Ca^2+^ (mmol/L)	1.96±0.32
P^3+^ (mmol/L)	1.67±0.71
pkt/v	1.3369±0.4425
rkt/v	0.5125±0.5017
tkt/v	1.8626±0.2144

Data are reported as (mean±SD) or as indicated. BMI: body mass index; CCI: Charlson comorbidity index; NEUT: absolute neutrophil count; LYMPH: absolute value of lymphocyte; HGB: hemoglobin; ALB: serum albumin; CK-MB: creatinine kinase MB; TC: cholesterol; GLU: blood glucose; Ca^2+^: blood calcium; P^3+^: blood phosphorus; pkt/v: peritoneal urea clearance index; Kt/v: urine urea clearance index; tkt/v: total urea clearance index.

Among the 110 patients with early-start PD, 85 patients (77.3%) continued to undergo PD, 20 patients (18.2%) died, 4 patients (3.6%) switched to HD, none of the patients (0%) underwent renal transplantation, and one patient (0.9%) was lost to follow-up. Of the 20 patients who died, 10 patients died of heart failure, three patients died of stroke, two patients died of myocardial infarction, two patients suffered sudden death, two patients died of pneumonia, and one patient died from a car accident. Of the 4 patients who switched to hemodialysis, one patient had refractory bacterial peritonitis, one patient had fungal peritonitis, and two patients had high peritoneal transport. The patient who was lost to follow-up returned to the hospital one time per month for two times, thereafter follow-up was ended, with no answer of phone calls. The number of withdrawals was 25, and the total withdrawal rate was 22.7%.

### Overall survival rate and technical survival rate

For the early-start PD patients, the 1-, 2-, and 3-year survival rates were 90, 81.8, and 81.8%, respectively, while the 1-, 2-, and 3-year technical survival rates were 89.1, 79.1, and 79.1%, respectively.

### Comparison of survival rates of early-start PD patients with different primary diseases

**Figure 1:**
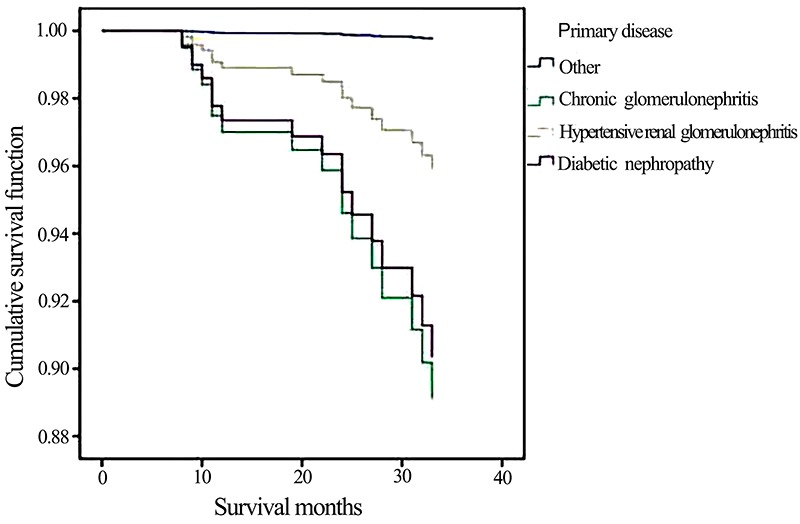
Comparison of survival curves of urgent-start peritoneal dialysis patients with different primary diseases.

The survival rates were compared among groups based on the primary diseases that induced ESRD: chronic glomerulonephritis group, diabetic nephropathy group, hypertensive renal damage group, and other primary diseases group. The results revealed that the survival rate was significantly lower in the chronic glomerulonephritis group (P=0.003), hypertensive renal damage group (P=0.022), and diabetic nephropathy (P=0.008), compared to the other primary diseases group (Figure 1).

### Charlson comorbidity index and survival rate

**Figure 2:**
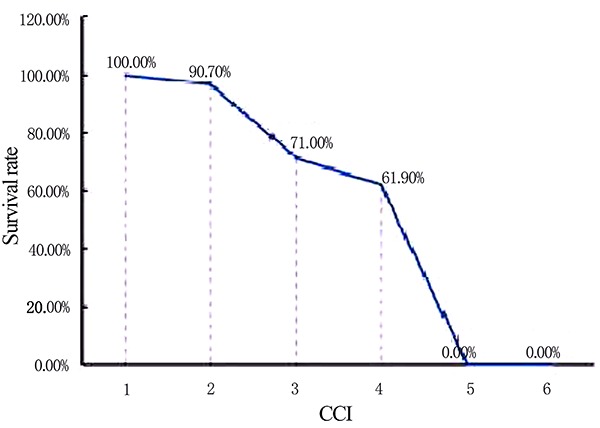
Comparison of 3-year survival rates of urgent-start peritoneal dialysis patients with different Charlson comorbidity index (CCI).

The Jonckheere-Terpstra test was used to compare the 3-year survival rate among patients with different CCIs. The results suggested that the higher the patient's CCI score, the lower the survival rate (P=0.000, Figure 2).

### Analysis of survival-related factors

#### Univariate analysis

With survival time and outcome as the analysis variables, factors that might affect the survival of patients with early-start PD were analyzed using univariate COX regression analysis. Some measurement indicators (age, BMI, hemoglobin, and serum albumin) were converted into categorical variables based on clinically and commonly used cut-off values, and some measurement indicators (systolic blood pressure, diastolic blood pressure, absolute neutrophil count, absolute lymphocyte count, creatine kinase-MB [CKMB], total blood cholesterol, blood glucose, blood calcium, blood phosphorus, pkt/v, rkt/v, and tkt/v) were converted into categorical variables based on quartiles. The results revealed that differences in the CCI index, absolute neutrophil count, and albumin level among early-start PD patients with different survival statuses were statistically significant (P<0.05, for all; Table 2).

**Table 2:** Univariate COX regression analysis of survival-related factors in early-start peritoneal dialysis patients.

	B	SE	Wald χ ^2^	P value	RR	95%CI	
						Lower Limit	Upper Limit
Gender	0.047	0.450	0.011	0.917	0.954	0.395	2.304
Age	−0.402	0.559	0.516	0.472	0.669	0.224	2.002
BMI	0.421	0.218	3.735	0.053	1.524	0.994	2.335
Systolic Blood Pressure (mmHg)	0.049	0.204	0.058	0.810	1.050	0.704	1.568
Diastolic Blood Pressure (mmHg)	0.323	0.230	1.973	0.160	1.381	0.880	2.168
Primary Diseases	0.159	0.270	0.347	0.556	1.173	0.691	1.991
CCI	0.924	0.180	26.267	0.000	2.520	1.769	3.588
NEUT (×10^9^/L)	0.424	0.215	3.901	0.048	1.528	1.003	2.326
LYMPH (×10^9^/L)	−0.054	0.031	3.136	0.077	0.947	0.892	1.006
HGB (g/L)	0.361	0.469	0.593	0.441	1.435	0.572	3.597
ALB (g/L)	2.000	0.748	7.153	0.007	7.389	1.706	32.001
CKMB (ng/mL)	0.100	0.213	0.222	0.638	1.106	0.728	1.679
TC (mmol/L)	−0.043	0.211	0.041	0.840	0.958	0.633	1.450
GLU (mmol/L)	−0.022	0.213	0.010	0.919	0.979	0.644	1.487
Ca^2+^ (mmol/L)	−0.275	0.195	1.987	0.159	0.760	0.519	1.113
P^3+^ (mmol/L)	−0.133	0.206	0.417	0.519	0.876	0.585	1.311
pKT/V	−0.465	0.216	4.655	0.051	0.628	0.412	0.958
rKT/V	0.326	0.208	2.475	0.116	1.386	0.923	2.082
tKT/V	0.326	0.208	2.475	0.116	1.386	0.923	2.082

BMI: body mass index; CCI: Charlson comorbidity index; NEUT: absolute neutrophil count; LYMPH: absolute value of lymphocyte; HGB: hemoglobin; ALB: serum albumin; CK-MB: creatinine kinase MB; TC: cholesterol; GLU: blood glucose; Ca^2+^: blood calcium; P^3+^: blood phosphorus; pkt/v: peritoneal urea clearance index; Kt/v: urine urea clearance index; tkt/v: total urea clearance index; RR: risk ratio.

#### Multivariate analysis

Factors that were statistically significant (P<0.05) in univariate analysis, such as CCI, absolute neutrophil count, and serum albumin, and factors that were clinically significant (age, primary disease, and hemoglobin) were analyzed using the multivariate Cox's proportional hazard regression model. The results revealed that an age ≥65 years old at the beginning of dialysis (RR=11.164, 95%CI: 1.271–98.034), diabetic nephropathy (RR=47.099, 95%CI: 3.443–644.261), hypertensive renal damage (RR=18.024, 95%CI: 1.363–238.343), chronic glomerulonephritis (RR=41.953, 95%CI: 3.105–566.915), CCI (RR=6.938, 95%CI: 3.339–14.418), hemoglobin (RR=4.307, 95%CI: 1.121–16.542), and serum albumin (RR=10.333, 95%CI: 2.009–53.137) were independent risk factors that affected the survival rate of early-start PD patients. The mortality in patients ≥65 years old was 11.164 times patients <65 years old. The mortality in of patients with diabetic nephropathy, hypertensive renal damage, and chronic glomerulonephritis was 47, 18, and 41 times that of patients with other primary diseases, and the mortality increased 5.9 times when the CCI increased by one point. Furthermore, the mortality of patients with hemoglobin of <90 g/L was 4.3 times that of patients with hemoglobin ≥90g/L. Moreover, the mortality of patients with serum albumin levels <30 g/L was 10.3 times that of patients with serum albumin levels ≥30 g/L (Table 3).

**Table 3:** Multivariate COX regression analysis of survival-related factors in early-start peritoneal dialysis patients.

	B	SE	Wald χ^2^	P value	RR	95%CI	
						Lower limit	Upper limit
Age	2.413	1.109	4.737	0.030	11.164	1.271	98.034
Diabetic nephropathy	3.852	1.335	8.331	0.004	47.099	3.443	644.261
Hypertensive renal damage	2.892	1.317	4.818	0.028	18.024	1.363	238.343
Chronic glomerulonephritis	3.737	1.328	7.912	0.005	41.953	3.105	566.915
CCI	1.937	0.373	26.943	0.000	6.938	3.339	14.418
NEUT (×10^9^/L)	0.359	0.242	2.200	0.138	1.432	0.891	2.301
HGB (g/L)	1.460	0.687	4.523	0.033	4.307	1.121	16.542
ALB (g/L)	2.335	0.835	7.813	0.005	10.333	2.009	53.137

CCI: Charlson comorbidity index; NEUT: absolute neutrophil count; HGB: hemoglobin; ALB: serum albumin; RR: risk ratio.

## Discussion

Patients on urgent-start PD are a mix of those with unrecognized advanced CKD and those whose CKD was recognized but worsened unexpectedly (5). Thus, we thought that early-start PD was better suited for those patients, which were included in this study, although all the 110 patients required dialysis within 72 h. Taking into account that the greatest cause of failure of PD is the high incidence of complications, such as dialysis leakage, bleeding, and short-term peritonitis after PD catheterization (4,8), early international PD guidelines considers that 14 days after catheter insertion is a relatively ideal time of initiation of PD (3,9). Thus, the early use of PD is limited. With the continuous improvement of PD technology, such as the use of a Tenckhoff catheter and a closed liquid supply system with a Y connection, the improvement of catheterization, and the application of automatic PD, some studies have confirmed that early-start PD is safe and reliable for patients with ESRD (10,11). The differences in the incidences of short-term catheter-related complications and peritonitis, survival rate, and technical survival rate between early-start PD and regular PD were not statistically significant (12). Compared with early-start HD, early-start PD can reduce the incidence of bacteremia, but the differences in short-term catheter-related complications and survival rate between early-start PD and early-start HD were not statistically significant (10,13). However, these studies were based on PD within 14 days, and there are few reports on immediate treatment after catheter insertion. If the condition requires immediate dialysis in order to avoid catheter-related complications and the reduction in technical survival rate, HD is often selected for transitional therapy after PD catheter insertion. This increases the risk of cross infection and the cost of medical treatment.

A study revealed that in the first 90 days, in case of urgent-start dialysis, the estimated cost for each patient was US$16,398 for PD and US$19,352 for HD (14). Therefore, urgent-start PD is a cost-saving method. The Yunnan province is located on the southwestern border of China, which has the largest number of ethnic minorities in China, and most of the populace dispersedly lives in remote and scattered areas. Moreover, the living environment is poor and education level is low, leading to low awareness of self-care and health risk. Therefore, the visiting rate is low, and patients have multiple and severe complications, including serious acid-base disturbances, electrolyte disturbance, high volume load, etc., during a hospital visit. Hence, these patients often need to immediately undergo dialysis. Due to causes such as the underdevelopment of the economy in Yunnan, limited medical insurance, and the poverty of patients, it is difficult to use HD for transitional therapy after PD catheter insertion, and immediate dialysis within 24 h after catheter insertion is suitable.

The technical survival rates of patients in this study was roughly similar to that in other areas with relative satisfactory PD development (10,13), and was higher than that in partial areas (15). This suggests that the technical survival rate in patients who underwent PD within 24 h after catheter insertion in our center was similar to that in developed domestic areas and some developed countries. In all patients with early-start PD in our center, catheter insertion was conducted by experienced nephrologists through surgical incision, and the peritoneum was tightly sutured to reduce the incidence of leakage and bleeding. Since experienced nephrologists are more familiar with the patient's condition and can pay attention to local details, they could improve the quality of PD catheter insertion, and accordingly increase the success rate of catheter insertion and technical survival rate (16). This may be correlated to the high technical survival rate of patients in our PD center.

Although early-start PD has many advantages, its prognosis remains a concern that must be given attention to and improved. A study has revealed that the prognosis of PD patients may be closely correlated to the age of PD beginning, primary disease, residual renal function, peritoneal transport function, anemia degree, nutritional status, cardiovascular and cerebrovascular diseases, infection and other complications, economic factors, living habits, and psychological factors. In recent years, due to the attention given to factors that affect the prognosis of PD patients, the survival rate of PD has increased (17,18). Studies in some economically developed areas revealed that the 1-, 2-, and 3-year survival rates were 90, 95, and 81%, respectively (12,19). In the present study, the rates were basically consistent with those studies. These results revealed that PD within 24 h after catheter insertion did not affect the survival rate of patients.

Old age, diabetes, and low baseline serum albumin are predictive factors for poor prognosis in PD patients. A study revealed that the 5-year survival rate in middle-aged and elderly PD patients was significantly lower than that in non-elderly patients, and this was the lowest in advanced age patients. The reason may be the increase in the number of complications with the increase in age of PD patients (20). Based on our results and those of others, CCI (21), age (20), malnutrition (22,23), and diabetic nephropathy (24,25) can be considered risk factors for predicting survival of PD.

Although most of the patients in our center come from various parts of Yunnan Province, there are still some limitations in the present study: lack of extensive, comprehensive, and multi-center data and the possibility of single-center specificity cannot be excluded. Furthermore, some indicators with defined clinical significance (such as education, economic level, residual renal function before dialysis, peritoneum transport, and peritonitis) were not analyzed. In addition, the sample size was too small and there was lack of comparison between emergency PD and planned PD and between emergency PD and HD. Therefore, multi-center joint studies with larger samples are required to confirm these results.

In summary, early-start PD can be used as a safe choice. However, patients are not recommended for treatment with urgent dialysis when they are in critical condition and CCI is relatively high. Although it did not affect the technical survival rate, it can affect the quality of life of these patients. In addition, the principle of active prevention and treatment of complications should be observed throughout the whole process of PD treatment, and attention should be given to follow-up in order to timely discover and correct the unfavorable factors of PD, and improve the survival rate and quality of life of PD patients.
